# The extent of task‐sharing implementation as a strategy to expand abortion services in Colombia

**DOI:** 10.1002/ijgo.12999

**Published:** 2020-07-31

**Authors:** Maria M. Vivas, Salomé Valencia

**Affiliations:** ^1^ Oriéntame Clinics Bogotá Colombia

**Keywords:** Abortion services, Accessibility, Colombia, Decentralization, Induced abortion, Primary healthcare, Task‐sharing, Women's health services

## Abstract

**Objective:**

To analyze the extent to which task‐sharing to midlevel providers has been implemented as a strategy to increase access to abortion provision in Colombia, and examine the factors that have affected decentralization of services.

**Methods:**

We conducted a case study based on the World Health Organization's 2015 guideline: *Health Worker Roles in Providing Safe Abortion Care and Post‐abortion Contraception*. Documentation was collected on the standard and epidemiological landscape of abortion in Colombia, followed by semistructured discussions with groups and individual stakeholders.

**Results:**

Task‐sharing as a distinct policy to increase access to abortion services has not been implemented in Colombia. However, role distribution toward nonspecialist physicians has been used as a strategy to ensure access. Other professionals, such as nurses, have limited tasks in abortion care despite evidence to support a more expanded role.

**Conclusion:**

The implementation of task‐sharing as a strategy to increase access to safe abortion services in Colombia is influenced by a wide range of factors and, although it is not policy, nonspecialist and diverse healthcare professionals supervise abortion care. Knowing the evidence‐based guidelines to safely and successfully include other healthcare professionals in abortion provision is a fundamental step in implementing this strategy.

## Abstract 2

Task‐sharing for abortion provision does not exist as policy in Colombia. However, the C‐355 ruling that decriminalized abortion in 2006 paved the way for distribution of tasks to nonspecialist physicians and other healthcare professionals, and the decentralization and simplification of services.

## 1 Introduction

In 2015 the World Health Organization (WHO) issued guidelines that recommend including a broad range of healthcare professionals in abortion care.^1^ This is an important strategy to ensure access to services, as well as an important tool to refute the misunderstanding that abortion is a complicated and unusual service that belongs in complex healthcare facilities controlled largely by specialist doctors acting as gatekeepers.

This “task‐sharing” strategy helps to normalize abortion, focuses services on women, actively involves community members in health care, and contests stigmatization. Shifting tasks to midlevel providers requires the availability not only of trained personnel, adequate facilities, and established referral systems, but also clear guidelines that describe and match personnel tasks to the appropriate complexity level of service.

The present paper describes a case study conducted in Colombia where abortion is decriminalized and legal conditions are set for women to request services and for providers to offer them. That said, since abortion became partially decriminalized in 2006,^2^ access to abortion services has been uneven throughout the country. There are still many barriers that prevent women from exercising their sexual and reproductive rights in Colombia.

The country has profound inequalities that, historically, have halted access to basic services and the full exercise of rights. Gaps in health indicators exist between geographical areas (rural or urban), and socioeconomic and/or educational levels; for example, lack of access to comprehensive sex education and belonging to the lowest level of wealth predict poor access to contraception.^3^ Inequities persist despite broad health system coverage, with more distant areas lacking trained personnel and availability of supplies.^4^ Colombia is divided into different departments (*departamentos*) that are administrative and political units inscribed into central government. Analysis of maternal mortality by social determinants of health shows that women living in departments with the highest proportion of unmet basic needs have a 1.72 times higher risk of death; the risk also increases in municipalities with predominantly indigenous and Afro‐descendant populations.^5^

The healthcare system in Colombia is weak in many ways, including the lack of processes and systems to monitor abortion provision. Accurately estimating the number of induced abortions in Colombia is a major challenge given the lack of a functional official reporting system and the unknown numbers of clandestine abortions still practiced. Both phenomena are linked to stigmatization of abortion services, which is prevalent throughout the country.^6^

Availability of abortion services continues to be poor. In 2008, only 11% of all health facilities in Colombia provided legal abortion services—most of them concentrated in urban areas and in the wealthiest departments of the country.^7^ The consequence of socioeconomic inequalities and lack of access to safe abortion services is that a higher proportion of women with lower socioeconomic status and low levels of education do not receive health care after an abortion even though they need it. It is estimated that 50% of poor rural women who suffered from complications after an unsafe abortion did not receive postabortion care.^7^

Although there is a lack of available services in peripheral areas of the country, there are successful experiences, especially in the main cities. These two contrasting scenarios happen within the same regulatory framework; therefore, successful experiences in one place could be replicated in others. The aim of the present study was to analyze the extent to which task‐sharing to midlevel providers has been implemented in Colombia, the factors that contributed to the decentralization and simplification of services, the obstacles experienced in doing so, and how it has impacted access to abortion in the country.

## 2 Materials and Methods

This case study comprised two phases: (1) a review of the literature and; (2) discussions with key informants. The purpose was to analyze the factors, and their interactions, that were associated with implementing task‐sharing in abortion provision as a strategy to increase access to services in Colombia. Institutional Review Board approval was not required for this study.

The first phase consisted of collecting documentation on the standard and epidemiological landscape of abortion in Colombia. It included the most relevant changes and advances in decriminalization, as well as public policies related to service provision and the implementation of task‐sharing. The literature review was based on four categories related to these objectives using academic literature databases (PubMed, LILACS) and grey literature from government and expert institutions in the field. We also gathered documentation on the distribution of tasks among healthcare professionals, and facility requirements—particularly sexual and reproductive health services.

The second phase consisted of conducting individual and group discussions that covered different topics, including description of the current policy on task‐sharing; strategies employed for implementation; coalitions or collaborations involved in implementation; guidelines and practices; capacity, infrastructure, and resources; user experience (health workers and women); and monitoring and evaluation/research. A set of topics were selected, with the advice of an expert, to better match the profile of participants. The study was conducted in Bogotá, Colombia, and a total of 16 participants with varied profiles representing different institutions provided information in July and August 2018 (Table 11).

**Table 1:** Study participants

Type of institution	Number of participants	Current position
Government		
MINSALUD	3	Coordination of the sexual and reproductive health group, a medical doctor, and a lawyer
Providers		
Private provider	3	Medical director and nurse coordinator/Executive Director
Public provider	2	Medical providers
Provider in region	1	Medical coordinator of ESAR
Medical associations		
FECOLSOG	1	Academic coordinator
ANEC	1	Member of the organization
NGOs		
Profamilia	1	Medical Director
La Mesa por la vida y la salud de las mujeres	2	Coordinator, lawyer
Educational institution		
Uniandes	1	Professor, faculty of medicine

Abbreviations: MINSALUD, Ministry of Health and Social Protection; FECOLSOG, Colombian Federation of Obstetrics and Gynecology; ANEC, National Association of Nurses.

## 3 Results

### 3.1 Background: Distribution of tasks in sexual and reproductive health care

The distribution of tasks in sexual and reproductive health in Colombia precedes the decriminalization of abortion in 2006. Norms already specified the extent of duties regarding other sexual and reproductive healthcare services for different health workers. Furthermore, the decentralization of tasks from specialist providers is not exclusively related to reproductive health or abortion. Tuberculosis and mental health care are among other services that include other nonphysician professionals in service delivery.

Currently, and according to regulations, nonspecialist physicians are able and allowed to perform gynecological assessment and specific screening; diagnosis and prevention of sexually transmitted infections (STIs); contraceptive counseling; and delivery of contraceptive methods, including insertion and removal of implants and intrauterine devices. General practitioners must also be trained to identify mental health risk factors and victims of gender violence and attend to them accordingly. These two tasks are important because they are the basis under which two of the legal exceptions allowing access to abortion in Colombia are certified.

Nurses can provide direct healthcare services in primary care institutions and programs and have the mandate to prioritize the most vulnerable populations. The Ministry of Health and Social Protection (MINSALUD) has issued a series of protocols to clearly describe nurses’ activities,^8^ with specific mention of sexual and reproductive health‐related activities, such as preconception and prenatal care, contraception counselling, emergency contraception, diagnosis and prevention of STIs, and screening of neoplasic diseases such as breast and cervical cancer. Nursing assistants administer medications and perform certain procedures according to the doctor's prescription.

Psychologists and social workers can identify and conceptualize relevant problems and perform psychological analysis of individual and social relevance. They can also perform evaluation and diagnosis, and design and implement health promotion, prevention, and intervention programs. Pharmacists manage and provide information to customers on pharmaceutical products.

Task‐sharing already existed before the decriminalization of abortion. Several tasks related to other sexual and reproductive services were already assigned to midlevel healthcare professionals as part of their roles. Although these profile descriptions, written before 2006, did not include anything related to abortion services, the intersection between the ruling that depenalized abortion and these norms creates an opportunity to build a comprehensive route of care for abortion.

### 3.2 Regulation for abortion care services

In May 2006 the Constitutional Court of Colombia issued the C‐355/2006 ruling that decriminalized abortion under three circumstances: when a woman's health or life is in danger; when there is a fetal malformation incompatible with life; and when the pregnancy is due to sexual violence or unconsented insemination. The C‐355 ruling also established service provision requirements (Box 1).^2^ In 2005, before the decision of the Constitutional Court was announced and foreseeing the upcoming partial decriminalization, the Ministry of Health initiated a process to formulate a regulatory framework for safe abortion care in Colombia. The first legal abortion care protocol^9^ was drafted based on the guidance published by the WHO in 2003 and was adopted by the 4905/2006 Resolution.^10^ Subsequently, Decree 4444 of 2006 was issued to regulate sexual and reproductive health services and established norms of availability, financing, conscientious objection, and sanctions in case of the imposition of barriers to access.^11^ However, the State Council derogated both resolutions owing to an error in the process and as part of the antichoice strategy. Nevertheless, determined providers and feminist collectives insisted on the implementation of service provision, arguing that C‐355/2006 contained all requirements needed to provide services.

Box 1 **Standards of lawful abortion in Colombia according to C‐355/2006.**

1

**Table:** 

The right of abortion is recognized under three circumstances and only those requirements clearly defined in C‐355/2006 can be requested: The woman's life or health is at risk, certified by a physician. When there is a risk to mental health, a doctor or psychologist can issue a certificate. When there are fetal malformations incompatible with life outside the uterus, certified by a physician. When the pregnancy is the result of criminal acts, such as rape, incest, or nonconsensual insemination, officially reporting the crime.
Legal abortion services must be offered by public and private healthcare services in all levels of the health system.
Women under 14 y of age can express their will and no third‐party authorization is required.
There is no gestational age limit.
The Constitutional Court recognized an individual's right to conscientious objection for those directly providing services (physicians and nurses). It cannot be exercised by administrative personnel, institutions, or judges.

In the same year, the National Council for Social Security in Health issued Agreement 350/2006 ruling that all abortion procedures including manual vacuum aspiration (MVA), medical abortion, dilatation and evacuation (D&E), and fetal demise should be provided within the public healthcare system (*Plan Obligatorio de Salud*).^12^ In 2007, the Ministry of Health stated that public facilities must guarantee the number of healthcare providers available to offer abortion services.^13^ Subsequently, a series of guidelines were produced.^14^, ^15^, ^16^, ^17^ These guidelines explain the concepts and current legislation for healthcare staff and administrative personnel. For service provision, they specify that at least one nonspecialist physician, a counselor, and a nurse should be involved. In addition, legal standards were further developed to improve the process by eliminating barriers, increasing protocol adherence, and ensuring quality of services at all levels of care (Table 22).

**Table 2:** Regulatory framework for abortion services in Colombia

Title	Year	Aim
Decree No. 1011 of 2006. Mandatory System of Quality Assurance of Health Care of the General System of Social Security in Health (SGSSS)^a^	2006	Healthcare service providers must comply with the unique service quality assurance system, meaning that they must fulfill basic conditions of technological and scientific capacity, equity and financial adequacy, and technical‐administrative capacity. All healthcare services must comply with the same quality assurance system
Decree 4444 of 2006. Ministry of Social Protection by which the provision of services in sexual and reproductive health is regulated^b^	2006	The Decree “which regulates the provision of sexual and reproductive health services” regulated the provision of legal abortion. It included eight articles with the technical conditions for the social security health system to ensure women's access to a legal abortion, such as levels of complexity and financing conditions. The 4444 Decree was later derogated by a form procedure and has not been reestablished
Resolution 4905/2006 of the Ministry of Social Protection by which the Technical Standard for the Voluntary Interruption of Pregnancy is adopted^a^	2006	The technical norm is adopted to provide services for the voluntary interruption of pregnancy. In addition, the unique codification of procedures is added. The provisions herein apply to entities promoting health services, administrators of the subsidized regime, adapted entities, prepaid medicine entities, among others
Circular 31/2007 of the Ministry of Social Protection^a^	2007	Information on the provision of legal abortion (professionals willing to provide abortion services, the barriers and complications, among other information, updated and available)
Agreement 350/2006 of the National Social Security Council^a^	2006	The attention of legal abortion is included in the public healthcare system
Decree No. 3039/2007 adopting the National Public Health Plan for 2007–2010^b^	2007	Established rules to guarantee abortion service provision in the three exceptions determined by the C‐355/2006 Sentence, determined quality criteria, and protected women's sexual and reproductive health and rights
Circular No. 068 of 2008 regarding minors under 14 y of age^a^	2008	“Any protective measure that deprives any 14‐y‐old child of giving abortion consent is considered not only as unconstitutional, but as counterproductive to the effectiveness of her fundamental rights and to the defense of her legitimate superior interest, for being openly contrary to human dignity”
Resolution No. 769/2008 for cases of voluntary interruption of pregnancy requested by adolescents^a^	2008	Adolescents have the possibility of making decisions regarding their body and their health, and not to put life at risk
Law 459 of 2012 regarding the Protocol of Comprehensive Health Care for Victims of Sexual Violence^a^	2012	The healthcare sector committed to guarantee women's rights, by detecting cases of sexual violence, preventing such cases, and offering, quality and comprehensive care, among others
Circular No. 003 of 2013 of the National Superintendence of Health^c^	2013	“Establishes that all health service providers: (a) are obliged to provide abortion services to women who are covered by any of the exemptions; (b) are prohibited from generating service barriers and obstacles; (c) must guarantee an adequate number of health care providers who can performed abortion procedures; (d) must guarantee the attention of the woman in a timely manner (5 d after the consultation); and (e) must implement C‐355 of 2006”
Resolution No. 1441 of 2013 of the Ministry of Health and Social Protection^a^	2013	“It defines the minimum standards and enabling conditions that must be fulfilled by health care providers, independent health professionals, special patient transportation services and entities with a social purpose other than the provision of health services that, by requirement of its activity, provide exclusively low complexity services and specialized consultation, and do not include hospitalization or surgical services”
Resolution No. 1841 of 2013 of the Ministry of Health and Social Protection. The National Public Health Plan 2012–2021^a^	2013	The 10‐y National Public Health Plan aims to “guarantee the highest level of Sexual and Reproductive Health through: (a) the promotion of sexual and reproductive rights and gender equality, and (b) prevention and comprehensive, humanized and high quality care, from a human rights perspective, with gender and inclusive approaches, through the articulation of the different sectors that impact those social determinants related to sexual and reproductive rights”
Resolution No. 5.521 of 2013 updates the National Health Program^a^	2013	It includes the attention for legal abortion, which had already been established. It includes misoprostol in the National Health Plan, for the evacuation of the uterine cavity in the following cases: dead fetus in the second and third trimesters of pregnancy; early pregnancy failure with gestational age less than 22 wk; and voluntary termination of pregnancy under the three approved circumstances

a Regulation in force.

b Regulation derogated/annulled.

c Regulation partially annulled in 2016.

Facilities must also comply with specific requirements based on regulation according to their level of complexity.^18^ In a first‐level facility, a nonspecialist physician trained to provide abortion services and to identify situations that require prompt referrals to higher levels, if necessary, must be available.^14^ Specifically, they must be able to provide abortion by MVA up to 15 weeks and by medication up to 10 weeks of pregnancy. They must also be able to identify, stabilize, and refer any patient with complications that cannot be managed. Given that most abortions happen during the first trimester, the majority can be performed in a facility with the lowest level of complexity. A secondary‐level facility must have the same services as a first‐level facility. For procedures over 15 weeks, hospitalization should be available if necessary, at least with a referral system in place. Likewise, secondary‐level facilities must be able to handle complications related to abortion. All tertiary‐level institutions must have the personnel and physical capacity to perform abortion procedures under any circumstances allowed by law and to handle complications. University hospitals should promote training for professionals during clinical training rotations to ensure that enough personnel are trained to provide abortion services.

Since the decriminalization of abortion in 2006, several protocols have been developed—all of them specifying that general practitioners must be able to provide abortion and postabortion care services at the primary‐care level for the most common cases of abortion. Because the constitutional law—the highest‐level ruling in the country—was specific about service provision, when some of the lower‐level regulations, specifically 4905/2006 resolution and decree 4444 of 2006, were derogated as part of the antichoice strategy, there were still clear guidelines that could not be subverted. Norms also stipulated that abortion services be available in public hospitals, and protocols specified the composition of the group of healthcare providers. These roles are also in accordance with previous sexual and reproductive health service regulations. In other words, regulation in Colombia allowed for decentralization of abortion from specialist care to include other cadres of professionals.

### 3.3 Implementation of task‐sharing for abortion provision in Colombia

Since task‐sharing is permitted in Colombia, at least to some extent, and protocols and guidelines characterize MVA and medical abortion as procedures that can safely happen in primary health care, one would expect that all abortions performed before 15 weeks take place in primary healthcare centers and are done by nonspecialist doctors. However, other factors, such as how the route of care is conceived from the beginning, whether it is a public or private facility or a center dedicated to sexual and reproductive health care, and the political will to include other professionals deter inclusion of nonspecialist physician health workers.

The level of complexity and how the route of care is designed contribute to task‐sharing. Discussion participants who work in dedicated sexual and reproductive primary and secondary private healthcare centers stated that decreasing the level of complexity for abortion services was a priority from the moment the route of care was designed and the steps that comprise an abortion service were developed with workers’ roles in mind. Discussion participants from public centers that are also tertiary‐level hospitals considered that their main role was to support service provision in more complicated cases (more than 15 weeks of gestational age with comorbidities or complications). However, in these tertiary‐level hospitals, all abortions are performed by specialist doctors regardless of gestational age or presence of complications.

Political will from different stakeholders is also fundamental to the inclusion of a wider range of providers in abortion care. Supporters of this strategy were among those interviewed, including representatives from MINSALUD, Oriéntame clinics, the feminist collective “La Mesa,” and healthcare professionals committed to sexual and reproductive health care who have personally sought technical and legal training or have promoted the establishment of care routes at hospitals. Interviewees repeatedly mentioned that in the process of delegating abortion care from specialists to other types of professionals, support from MINSALUD and some local governmental institutions is key.

When asked what could be done to further delegate tasks to midlevel providers, respondents suggested involving healthcare insurers to establish comprehensive care routes in the services they administer. They also highlighted the importance of comprehensive and widespread training, not only in MVA and medical abortion techniques, but also in knowledge of regulations and the legal framework for professionals to help change preconceived ideas, mostly based on stigma, that prevent them from providing services. For instance, some respondents suggested that MINSALUD should train professionals on service provision guidelines at the primary level and provide technical assistance to these centers. La Mesa mentioned their role in economic and logistic support for technical training in MVA and medical abortion in different regions of the country, as well as legal training for abortion providers and administrative staff. Respondents mentioned the positive effect that providing abortion care services has on health workers, such as increased acceptability of abortion and change in attitudes and perceptions toward it. A central aspect that some participants discovered after developing abortion‐related skills and follow‐up of patients was satisfaction in being women's rights guarantors.

Currently, there is no specific policy that seeks to further expand the roles of healthcare professionals in any health service in Colombia beyond what has already been determined. During discussions, informants acknowledged that task‐sharing is a strategy that enables transfer of functions from specialist physicians to general practitioners; however, the role of nurses was considered in a tangential way, and the role of other healthcare providers or professionals was not even considered. This is clearly reflected in public hospitals where service provision is based on specialist physicians and there is resistance from hospital managers to change this scenario, in addition to the lack of clearly established abortion care routes.

In centers such as Oriéntame or Profamilia, nurses fulfill complex roles. For instance, in Oriéntame they conduct family planning counseling, insert implants and IUDs, and help guide procedures using ultrasonography. Despite this, to be considered safe, medical abortion and first‐trimester MVA can only be prescribed and performed by physicians.

Provision of abortion services by other midlevel providers, besides general physicians, cannot be considered without a change to the norm. When we asked the participant from the Colombian Federation of Obstetrics and Gynecology (FECOLSOG) their opinion about service provision by nurses, they explained that this was a source of concern owing to their lack of formal training to perform MVA or identify risk situations. However, they mentioned that if nurses were adequately trained, they would not oppose. It is worth mentioning here that medical doctors do not receive specific abortion training in medical school either.

A representative from MINSALUD believed that progress will be made in this regard, especially since abortion services were included in the Comprehensive Health Care Routes^19^ published in August 2018. This document describes the conditions necessary to ensure the integrality of care by the agents of the healthcare system (healthcare insurer, provider, and nonmedical professionals) and other sectors. In contrast, the same representative believed that, in many cases, expansion of roles is not necessary because there are specialist physicians available. Therefore, expansion would be in response to a need in territories with limited human resources capacity.

There was a perception among participants that political support for initiatives such as expansion of roles is deficient and not maintained over time. When routes have been established at first‐level hospitals, groups of 8–10 people including doctors, nurses, and psychologists or social workers are required. As such, the main resource needed for service expansion is trained personnel.

Interviewees mentioned repeatedly that the lack of flexibility in infrastructure regulation was a barrier to access. In hospitals, the investment that goes into infrastructure to provide abortion services is minimal because facilities are already suitable for carrying out other procedures, and only need to acquire medication and supplies to perform MVA. On the contrary, in the case of small healthcare centers or private offices, an important investment must be made to achieve quality standards in infrastructure. The government has invested in training but not in infrastructure because, according to the Colombian health model, this is the responsibility of healthcare insurers. Infrastructure barriers become almost insurmountable with the demands imposed by overregulation to set up healthcare centers owing to the difficulty in adopting those quality standards. Rural or remote areas are particularly affected.

Respondents reported that they could not see evidence of a relationship between the implementation of task‐sharing in general health care and in abortion care. In other words, what happens in other services regarding distribution of professional roles is not extrapolated to abortion care. This may be because sexual and reproductive health services, especially when focusing on abortion services, are treated differently, and face great resistance in society even among healthcare personnel. Task‐sharing is not evident in abortion care precisely because it is a strategy to increase access to abortion services.

Barriers to accessing abortion services interfere with the implementation of this strategy. Respondents mentioned that the ambiguous legal status and women's lack of information are factors that impact the distribution of roles. Although the Constitutional Court decriminalized abortion under three circumstances, and posterior jurisprudence elevates it to a right, abortion is still included in the penal code. On one hand it is a crime and on the other it is a fundamental right. On many occasions, the legal status (or lack of) of abortion is the framework from where health professionals conceptualize service provision, which contributes to the perception that abortion services are particular and complex. This “dual status” impacts on service provision and creates barriers, based on:

1. Healthcare providers’ lack of knowledge of the legal framework (i.e. not knowing principles such as the right to privacy and dignity), or absence of protocols for women's abortion care.
2. Restrictive interpretation of the legal framework, such as misuse of conscientious objection by healthcare professionals and institutions; request for legal and administrative requirements outside of those defined by the law; or requirement for assisted consent from representatives, guardians, or relatives of those aged 14 years or younger.
3. Deficiencies in the provision of the health service, such as lack of training of professionals to perform abortions, and discrimination and stigmatization against women.^20^

Misinterpretation of the law contributes to the notion that abortion is an abnormal service that must comply with a different set of rules than other services.

Participants did not identify any specific efforts that have been made to inform and educate health workers and women about the task‐sharing strategy, but considered that training and conferences for professionals, and information campaigns for civil society would offer information about who can provide safe abortion services.

### 3.4 Oriéntame

Oriéntame is a private women's clinic that exemplifies the enabling conditions that include a wider range of health workers in abortion care. It was created in 1977 to provide comprehensive services for postabortion care. Since 2006 it has provided abortion services in cases allowed by the Constitutional Court ruling C‐355. At its different sites, integration of midlevel professionals is sought to the extent that the regulation allows, and all healthcare professionals receive task‐specific training. The clinic has three types of outpatient facilities with different levels of complexity based on abortion method and gestational age: (1) facilities offering medical abortion only; (2) primary healthcare clinics where medical abortion and MVA are performed up to 10 and 15 weeks, respectively; and (3) secondary‐level care clinics for abortions after the first trimester.

Facilities that only provide medical abortion have different requirements in terms of infrastructure and equipment, and a nonspecialist physician and an auxiliary nurse manage the entire route of care. As the first step, the general practitioner provides preabortion and contraception counselling. A physical exam is then conducted and, if the patient is eligible for a medical abortion and wants to proceed, the general practitioner explains the rest of the process to her. An important aspect of services provided by Oriéntame is to include women as an integral part of their own abortion process. Therefore, mifepristone is administered by the nurse assistant at the clinic and misoprostol is taken at the place most convenient for the woman. The provider clearly explains what can be expected over the following days regarding adverse effects and warning signs and provides a brochure with relevant information and Oriéntame's contact numbers. A woman is not required to return to the healthcare service for a postabortion consultation unless she considers it necessary, she did not initiate a contraceptive method, or the doctor suspects an ectopic pregnancy.

In the clinics that perform medical abortion and MVA (up to 10 and 15 weeks, respectively) and D&E after 15 weeks, all doctors providing the services are general practitioners and the route of abortion care includes other nonmedical professionals, psychologists, social workers, medical doctors, and nurses who offer counselling. Nurses can provide postabortion contraception counselling and initiate any method of choice, both short‐ and long‐term methods. The clinics employ a referral system for complications, and procedures for monitoring and data collection and quality assurance that also guarantee compliance with the official requirements. In addition, each facility is supervised and visited by the health secretary.

Oriéntame has different means of ensuring that women know how to access services, including community outreach workers who provide information, pregnancy tests, and referrals to the clinics. In situations where women do not have easy access to technology and only have access to community‐based referral systems, these programs become fundamental; for example, as seen during the Venezuelan humanitarian crisis by connecting women without access to the internet with services. Table 33 shows the task distribution for abortion provision at Oriéntame.

**Table 3:** Task distribution for abortion provision at Oriéntame

Task	Pharmacist	Auxiliary nurse	Nurse	Psychologist/social worker	Nonspecialist doctor	Specialist doctor
Provision of information	X	X	X	X	X	X
Diagnosis of pregnancy and calculation of gestational age			X		X	X
Verify and certify that the case complies with C‐355/2006				X	X	X
Counselling and informed consent		X	X	X	X	X
Provision of surgical abortion					X	X
Provision of medical abortion		X	X		X	X
Contraceptive counseling			X	X	X	X
IUD, implant insertion and removal			X		X	X
Follow‐up when needed			X		X	

Oriéntame is a private institution where changes to service provision can be implemented quickly. Clinic directors understand how roles can be distributed, services simplified, and resources used appropriately. However, the regulations determining who can perform what tasks and the overregulation of infrastructure limit the implementation of a broader task‐sharing policy.

## 4 Discussion

Based on the discussions with experts and on Oriéntame's experience, expanding health worker roles in abortion provision increases access, reduces costs for both providers and women, and decreases waiting periods for women, especially those from vulnerable populations. To achieve role distribution toward nonspecialist physicians in abortion care, is it necessary to establish ample and clear legal frameworks from the moment that abortion is decriminalized to allow midlevel professionals to participate. In short, task‐sharing must be included as part of the decriminalization of abortion strategy and pushed from many fronts, not only by healthcare professionals but by feminists and advocates. In Colombia, the fact that task‐sharing for abortion provision was included in the highest‐level ruling on abortion has helped its implementation and prevented stagnation when lower‐level laws were derogated. In addition, norms and regulations must be implemented to their full extent, including roles and profiles established for other healthcare services.

Implementing task‐sharing for abortion provision has a circular nature. Midlevel professionals are involved in the delivery of other sexual and reproductive health services. Abortion should be treated as any other service. In turn, task‐sharing further normalizes abortion. In Colombia, the regulation that allows task‐sharing is not exclusive to abortion services but is used along with the C‐355 ruling and reinforced with specific protocols.

Barriers that need to be addressed include the widespread belief that specialized care is better‐quality care, and the notion that simplification of services is not necessary because specialists are available. Implementation of the strategy is further hindered by: (1) the perception that abortion is a difficult service to provide because of its technical and legal peculiarities; (2) the lack of technical capacity and knowledge of legal regulation by health providers; (3) the belief that delegating tasks to other healthcare professionals will lead to the replacement of specialist physicians.

Task‐sharing in abortion care is affected by factors that affect abortion in general, such as stigma, lack of information systems, and inequalities. Furthermore, lack of knowledge of the law and its limited interpretation contribute to the mystification of services. Finally, materialization of task‐sharing, to some extent, occurs in the route of care and in medical facilities. Therefore, overregulation to establish new services or to allow them to operate, especially those dedicated exclusively to abortion, is a barrier due to the cost of complying with infrastructure requirements. When healthcare managers design abortion services they must plan for the decentralization of tasks from the beginning.

In conclusion, although task‐sharing for abortion provision does not exist as specifically named policy in Colombia, it can be said that since 2006 a partial version of it has been implemented based on the service provision requirements stipulated by the C‐355 ruling. In addition, role definitions for healthcare professionals—different from specialist physician and established by MINSALUD before abortion decriminalization—have been incorporated into abortion services, expanding the cadre of professionals who can participate in abortion provision. However, this partial task‐sharing policy is not uniformly applied in different levels of complexity, and evidently does not include other professionals, such as nurses, who could perform additional abortion tasks and simplify services. WHO's evidence‐based task‐sharing recommendations prove that it can be done safely.

## Author Contributions

MV planned the desk review and data collection, identified interview participants, conducted data analysis, and wrote the manuscript. SV collected documents and performed desk review data analysis, interviewed participants, collected data, and prepared a preliminary analysis.

## Conflicts of Interest

The authors have no conflicts of interest to declare.
